# Editorial: Emerging arboviruses in the Americas: epidemiology, public health impact, and future preparedness

**DOI:** 10.3389/fpubh.2026.1907293

**Published:** 2026-08-10

**Authors:** André Ricardo Ribas Freitas, Marta Giovanetti, Luciano Pamplona de Góes Cavalcanti, Pedro María Alarcón-Elbal

**Affiliations:** 1 Faculdade São Leopoldo Mandic, Campinas, São Paulo, Brazil; 2 Oswaldo Cruz Institute, Oswaldo Cruz Foundation, Rio de Janeiro, Brazil; 3 Department of Science and Bio-Technology, Università Campus Bio-Medico di Roma, Rome, Italy; 4 Universidade Federal do Ceará, Fortaleza, Ceará, Brazil; 5 Departamento de Producción y Sanidad Animal, Salud Pública Veterinaria y Ciencia y Tecnologia de los Alimentos (PASAPTA), Facultad de Veterinaria, Universidad Cardenal Herrera-CEU, CEU Universities, Valencia, Spain

**Keywords:** arbovirus, chikungunya, dengue, emerging infectious diseases, epidemiology, Oropouche, yellow fever, Zika

Emerging arboviruses in the Americas cannot be understood as a single group of emerging pathogens, but as a heterogeneous, complex, and broad set of qualitatively different risks, each with its own epidemiological logic and public health implications. Three converging trends are reshaping this landscape: a worsening epidemiological situation, in which established threats reach unprecedented intensity; expansion into new ecological and geographic settings, as viruses move beyond their historical ranges and edge toward urban transmission; and the emergence of previously unrecognized or under-appreciated clinical forms. Confronting these intertwined dynamics demands an integrated, One Health response that connects human, animal, vector, and environmental surveillance—and that treats preparedness not as a single intervention but as a coordinated strategy matched to the distinct nature of each risk.

## A worsening epidemiological situation

The burden of arboviral diseases is intensifying rather than stabilizing. Dengue continues to reach unprecedented levels across the Americas, while geospatial clustering across Brazilian municipalities in 2024 highlights substantial fine-scale heterogeneity in transmission, underscoring the need for targeted and locally tailored control measures (Sena et al.). This challenge is not confined to endemic regions: screening of blood donors in Shenzhen, China, illustrates how arboviruses can silently move through global travel networks and establish risks far beyond their traditional areas of circulation (Zheng et al.).

Chikungunya provides a compelling example of the recurring epidemic waves that may characterize the coming decades. Twenty years after the devastating 2005–2006 epidemic, Réunion Island experienced another major outbreak in 2025. Our analysis estimated roughly 208 excess deaths among older adults during a 3-month period; the age-specific excess mortality rate reached 145.3 per 100,000 (95% CI: 125.5–165.0) with a case fatality rate (CFR) of 2.4%, substantially exceeding officially reported fatalities (1). National-level analyses from Brazil similarly demonstrate a significant burden of hospitalization and mortality associated with chikungunya infection (2). Together, these findings suggest that the true impact of chikungunya has been consistently underestimated. The Réunion experience serves as a warning for the Americas, where more than a decade has passed since the virus became established following its introduction in 2014–2015. Conditions favorable for a comparable resurgence may already be in place. A recurring lesson emerges across arboviral diseases: surveillance systems based primarily on laboratory-confirmed cases frequently underestimate the true burden of infection and disease.

Vectors remain central to this intensification. Several contributions in this Research Topic highlight the increasingly complex ecological and epidemiological factors shaping arboviral transmission. Studies range from the growing prevalence of pyrethroid resistance (kdr) mutations in Aedes aegypti populations in northeastern Argentina (Fay et al.)—threatening the long-term effectiveness of conventional vector control—to transcriptomic investigations of Wolbachia in this mosquito species (Mejías et al.), which reveal promising avenues for biological control interventions. Human factors also contribute substantially. A study assessing knowledge and attitudes toward chikungunya among healthcare workers in Chengdu, China, identified important awareness gaps that may delay recognition and diagnosis (Zhang et al.). Against this backdrop, climate variability—including the prospect of future El Niño events—is likely to amplify transmission risks and contribute to increasingly severe dengue seasons, alongside renewed activity of chikungunya, yellow fever, and Oropouche virus.

## Expansion into new environments

The most concerning trend emerging from this Research Topic is the continued geographic and ecological expansion of arboviruses. Yellow fever activity has intensified markedly, with 2025 recording the highest annual case count since 2020 and transmission extending into areas without a historical record of disease occurrence, including São Paulo State (3). Genomic investigation demonstrates the virus re-emerging across forest and periurban environments while advancing along forest edges toward densely populated, and often under-vaccinated, communities (4). Importantly, the prospect of yellow fever re-urbanization is no longer merely theoretical. Yellow fever virus has been detected and isolated from *Aedes albopictus* in an urban green area of São Paulo State (5). At the same time, *Aedes vittatus*—first recorded in the Americas in the Dominican Republic in 2019 (6) and now expanding across both insular and continental Caribbean regions—represents another potential bridge vector connecting sylvatic and urban transmission cycles (7). In this context, suboptimal vaccination coverage in newly affected areas remains the most important modifiable vulnerability.

Oropouche virus presents a parallel narrative of geographic expansion. Once considered largely confined to the Amazon Basin, the virus has increasingly demonstrated its capacity to spread beyond its historical range. Surveillance efforts in wild birds at rehabilitation centers in Florida are now evaluating whether competent vertebrate hosts exist in North America, an increasingly relevant question given the growing number of imported human infections (Newman and Hardman). Within Brazil, genomic surveillance recently documented the first imported case in Rio Grande do Sul, alongside enzootic circulation of *Culicoides paraensis*, signaling a tangible outbreak risk in one of the southernmost regions of the country (8). Additional studies further emphasize the importance of vigilance at the margins of known transmission zones. The detection of alphavirus circulation in Costa Rica (Valles-Morera et al.), together with the description of the first case of West Nile virus meningoencephalitis in southwest Michigan (Zou et al.), illustrates how arboviruses continue to establish themselves in new ecological settings, host populations, and vulnerable patient groups. Early recognition of such transitions remains critical for effective public health response.

## New and under-recognized clinical forms

As arboviruses expand into new territories, their clinical spectrum is also being redefined. Oropouche virus, historically regarded as a self-limiting febrile illness, has increasingly emerged as a pathogen capable of causing severe neurological disease and adverse congenital outcomes. Experimental evidence demonstrating enhanced virulence and neuroinvasive capacity of contemporary Oropouche virus strains in the AG129 mouse model provides biological support for these observations. Clinical reports describing neurological manifestations—including Guillain-Barré syndrome accompanied by bilateral visual impairment associated with an emerging reassortant lineage in Ceará (9)—further expand the recognized disease phenotype (Nobre Santos et al.). The possibility of congenital Oropouche syndrome has also gained increasing attention (Sansone et al.). A hypothesis initially raised within this Research Topic has since been reinforced by reports of newborns presenting with microcephaly following maternal infection in Brazil (10). These findings suggest that the public health implications of Oropouche virus may be substantially broader than previously appreciated. More broadly, mechanistic studies included in this Research Topic contribute valuable insights into arboviral pathogenesis. For example, evidence demonstrating that disruption of a glycosylation site in the Zika virus envelope protein attenuates viral replication provides important clues regarding determinants of flaviviral virulence and identifies potential targets for future intervention strategies (Wang et al.).

Chikungunya reinforces this theme from a different perspective. Rather than revealing a new clinical syndrome, recent evidence highlights a long-recognized but consistently underreported outcome: mortality. The findings from Réunion Island and Brazil clearly indicate that chikungunya-associated deaths remain substantially underestimated by conventional surveillance systems (1, 2).

## Toward preparedness

Several contributions in this Research Topic point toward the strategies needed to address future arboviral threats. Reviews examining current and emerging vector-borne disease risks (Hiscox et al.), together with analyses of knowledge gaps surrounding Caribbean arboviral vectors (Ali et al.), collectively support the adoption of integrated One Health approaches that recognize the interconnected roles of humans, animals, vectors, and the environment. As recently emphasized, closing both geographical and methodological gaps in arbovirus surveillance is essential for strengthening preparedness in an era of accelerating environmental change (11). Because case-based surveillance consistently underestimates the true burden of disease, complementary approaches are urgently needed. These include excess-mortality analyses and syndromic surveillance to identify otherwise unrecognized fatalities; integrated entomological and virological surveillance; monitoring of non-human primate mortality as an early warning system for yellow fever circulation; blood-bank screening as a sentinel tool for silent transmission; and minimally invasive tissue sampling to improve the investigation of arboviral deaths, an approach shown to be broadly acceptable in Brazil (12). These surveillance efforts must be accompanied by strengthened vaccination strategies. For yellow fever, as urgently called for by PAHO (3), vaccination coverage must be expanded across all populations exposed to sylvatic and rural transmission settings and increased until it attains at least the population-level protective threshold capable of preventing re-urbanization in newly affected areas. Likewise, broader implementation of available vaccines against dengue and chikungunya will be essential to reduce future disease burden. Together, enhanced surveillance, improved diagnostics, expanded vaccination, strengthened postmortem investigations, and timely public health interventions constitute the foundation of effective preparedness. Emerging arboviruses in the Americas—and increasingly worldwide—do not represent a single problem with a single solution. Rather, they comprise a complex and evolving constellation of threats that require tailored responses. Recognizing the distinct epidemiological, ecological, and clinical dimensions of each risk is the first step toward building the preparedness that the region urgently needs.
